# Benefits in Respect of Persons in Hospital

**Published:** 1913-02-15

**Authors:** 


### OUR NATIONAL INSURANCE ACT DEPARTMENT.

### XLIII.—Benefits in Respect of Persons in Hospital.

It is a fundamental principle of insurance that the insured person should not be allowed to benefit pecuniarily on the happening of the event insured against. Thus, in fire insurance the insurance company undertakes the liability of making good the loss actually occasioned by fire. The settlement of claim may, it is true, be made in money, but the cash payment is merely the amount of the assessed damages, and power is frequently reserved in fire insurance contracts to make good the loss by replacement of the articles destroyed. In a similar way life assurance is strictly regulated under an old Act of Parliament passed in the reign of George III., and commonly known as the Gambling Act, to the amount of the pecuniary insurable interest which the person insured has in the life assured.

This principle of insurable interest must also be taken into account in sickness and accident insurance, and has been given effect to in several ways under the National Insurance Act.

In the first place, it has to be premised that the object of sickness benefit is to mitigate the loss to an insured person owing to his being thrown out of employment by illness. It is manifest that if the benefit were made sufficiently attractive either in amount or by reason of the facility with which it could be claimed, it would provide an inducement to malingering. One way in which sickness benefit is limited in conformity with the principle of insurable interest is when the ordinary amount payable is more than two-thirds of the usual wage of the insured person. In this event it is possible for sickness benefit to be granted on a reduced scale, and for other benefits of equal value to be substituted in place of the reduction.

### No Benefits for Insured Persons in Hospital.

Another way in which the principle of insurable interest is made to operate is in the case of insured persons who are receiving treatment in hospital.

It is definitely laid down in Section 12 that "no payment shall be made on account of sickness, disablement, or maternity benefit *to or in respect of* any person during any period when the person to or in respect of whom the benefit is payable is an inmate of any workhouse, hospital, asylum, convalescent home, or infirmary, supported by any public authority, or out of any public funds, or by a charity, or voluntary subscriptions, or of a sanatorium or similar institution approved under the Act."

The contention here is that, as the insured person is receiving treatment in hospital—taking that term in its widest sense to include all the variations mentioned in the section—he is not in need of the benefits provided under the Act, and the logical consequence is that the benefits are not to become payable.

It may be noted in passing that the words of the Act above quoted are exceedingly wide, and embrace all possible cases. Thus it is to be noted that the prohibition is not only that the benefits shall not be paid *to* the insured person, but also *in respect* of the insured person; and, further, that the institutions named are not only those supported by public funds, but also those supported by voluntary subscriptions. Hence it is clear that the chief operative clause of the section prohibits the payments of the cash benefits payable under the Act to or in respect of insured persons who are receiving treatment in the voluntary hospitals.

### Exceptions to the Prohibition.

This principal operative clause is, however, modified by the second sub-section in such a way as to allow the payments of the benefits in certain circumstances.

The first case is the most important and the one that will most frequently occur, and therefore claims priority of attention.

When the insured person has dependants the benefit which would have become payable by his Society (or by the Insurance Committee if he be a Deposit Contributor) had he not been receiving treatment in hospital, is to be paid either in whole or in part for the relief or maintenance of his dependants. The manner in which the benefit is to be administered—whether in cash or otherwise—and to whom it is to be paid or distributed is to be determined by the Society or Insurance Committee responsible, after consultation, wherever possible, with the insured person.

It may here be pointed out that the expression "dependants" is interpreted by Section 79 to mean any person who, as ascertained by the Society or Insurance Committee, is dependent either wholly or in part on the earnings of the insured person. This is in sharp contrast to the definition furnished in the Bill as originally drawn, which would have limited the payment to relations within the following degrees—viz. husband, wife, parents, grandparents, children, grandchildren, step-parents, and step-children, and half-brothers and half-sisters.

There is no doubt that the definition under the Act is more reasonable than that originally suggested as it makes the determination of dependence to rest on fact, and allows the application of the benefit as was intended to the relief of those who are adversely affected by the incapacity of the bread-winner. We learn from a large Society that numerous claims have already been received for benefit on account of members in hospital, and that a very wide meaning is being given to the expression "dependants," so that anyone who can substantiate a claim to dependence is receiving the benefit.

The second case in which the benefit *has to be* paid in spite of the fact that the insured person is receiving institutional treatment is when the insured person is a member of an Approved Society and is receiving treatment in a sanatorium in accordance with the provision of the Act relating to sanatorium benefit. In this case, although he has no dependants, the Society has to pay the benefit to the Insurance Committee responsible for the sanatorium treatment, and the money is to be applied for the general purposes of that Committee.

### Agreements between Hospitals and Approved Societies.

Another case that may arise under the general prohibition of the section is that of an insured person who has *no* dependants receiving treatment in hospital. The special case where the treatment is for consumption in a sanatorium has already been dealt with, but in ordinary cases where the treatment is given to a member of an Approved Society who has no dependants in one of the voluntary hospitals the payment of the benefit to the hospital, or its retention by the Society, is a matter that is optional to the Society and depends upon any agreement that may have been made between the Society and the particular hospital.

If no agreement has been made with the hospital it must be presumed that the admittance of the member to the hospital ward was purely voluntary on the part of the hospital, and in consequence it can have no claim against the funds of the Society. If, on the other hand, an agreement has been made by a Society in the interests of its members for their reception and treatment in hospital, the terms of that agreement will have to be adhered to, and the money payable as benefit in the case of a member without dependants will have to be paid in whole or in part according to the terms of that agreement towards the maintenance of such person in hospital.

We are unable to say as yet what agreements, if any, have been entered into by the Approved Societies with hospitals for the treatment of members without dependants. It would certainly seem desirable, however, that the managers of voluntary hospitals should take early advantage of this particular section and communicate with the secretaries of the Approved Societies which have members resident in their district. It would be unreasonable on the part of the Society to take advantage of the fact that a member has no dependants when he is receiving institutional treatment to his advantage, thus retaining the benefit that would otherwise have become payable.

For this reason alone it would seem that there are good grounds for expecting a favourable response to overtures made to the Societies by hospital authorities. In addition to this it may be pointed out that Approved Societies are given power under Section 21 to subscribe or grant donations as they think fit to hospitals and other charitable institutions, and sums so expended are to be treated as payments on account of such benefits as the Insurance Commissioners may prescribe; that is to say, they would carry the State proportion of two-ninths in the case of men and one-quarter in the case of women.

If the Society has not entered into such agreements the sum which it would otherwise have been called upon to pay will remain in its funds, and thus go to benefit the members as a whole, unless the Society should think fit to make use of the money in the provision of any surgical appliances that the particular member may require or otherwise for his benefit.

It is to be noted that if the insured person is a Deposit Contributor and has no dependants the Insurance Committee responsible for the payment of benefits from his account has no power to make the payments to the hospital. In the same way, if the institution in which a member of an Approved Society is receiving treatment is supported out of public funds the benefit cannot be paid to that institution.

### Maternity Benefit not Payable to Dependants.

We have so far dealt with the relaxation of the prohibition of payments on account of sickness and disablement benefits. The prohibition does not only apply to these benefits but also to maternity benefit when this is payable on account of a woman who is herself insured. The rule providing for the payments of benefit to dependants does not apply to maternity benefit when this would be paid, except for the institutional treatment, in addition to sickness or disablement benefit. Maternity benefit is designed not as a relief for dependants but for the benefit of the mother herself. Thus the sickness benefit payable during the period of incapacity for work following confinement may be applied towards the maintenance of dependants, but not the maternity benefit of thirty shillings. The payment of the maternity benefit to the hospital is apparently left to the option of the Society, for the words used are: "Such sum *may* be paid to the hospital . . . . of which she is an inmate, in like manner as if she had no dependants." This is again a matter as to which it would seem desirable for the voluntary hospitals to enter into agreements with the Societies, and it should be noted that it is not only with women's Societies but also with men's Societies that agreements should be made. When both husband and wife are insured it is the husband's Society that pays the maternity benefit, and thus it is only by agreement with his Society that the payment can be allocated to the hospital in which his wife is an inmate.

We have seen it stated that the attitude of Societies towards the hospitals will largely depend upon the wishes of the members, and as the Societies are necessarily by their constitutions self-governing, it is apparent that the wishes of the members must ultimately prevail. If, then, the hospitals are to continue to receive insured persons they have every right to expect that the persons treated will support them in pressing for agreements with their Societies such as we have indicated.

The detail work in connection with the administration of benefits is absorbing all the energies of Society officials at the moment, and in the circumstances it is useless to expect them to take the initiative in regard to the agreements, but the concerted action of the hospital authorities in approaching the Societies would be a reasonable way to bring the matter to a successful issue.

